# Quality of Life and the Perceived Impact of Epilepsy in Children and Adolescents in the Eastern Province of the Kingdom of Saudi Arabia

**DOI:** 10.7759/cureus.20305

**Published:** 2021-12-09

**Authors:** Wesal Horaib, Razan Alshamsi, Najwa Zabeeri, Raidah S Al-Baradie, Moataza M Abdel Wahab

**Affiliations:** 1 Neurology, King Fahd Hospital of the University, Khobar, SAU; 2 Neurology, Imam Abdulrahman Bin Faisal University, Dammam, SAU; 3 Family and Community Medicine, Imam Abdulrahman Bin Faisal University, Dammam, SAU; 4 Neuroscience and Pediatric Neurology, Neuroscience Center, King Fahad Specialist Hospital, Dammam, SAU

**Keywords:** kingdom of saudi arabia, adolescent, epilepsy, pediatrics, anticonvulsants

## Abstract

Objectives

This cross-sectional study aimed to assess the perceived impact of epilepsy on children and adolescents and analyze its aspects.

Materials and methods

The study included patients with epilepsy aged between and two and 19 years old in three major hospitals in the Eastern Province of Saudi Arabia. Data were collected through an online survey.

Results

The mean score percentage of the quality of life (QOL) assessment was 65.6. This study showed better mean score QOL percentages in males (67), adolescents (65), patients with higher family income and socioeconomic status (64), and those living in Al Jubail (71). QOL was negatively associated with seizure frequency, the number of fears, problems, and concerns, and longer treatment duration. The most common concerns in children and adolescents with epilepsy are having/starting a relationship with others and what people at school will think if they have a seizure. The most common problems were lack of concentration and feeling short-tempered or grumpy. Continuing with education was the most common fear for the future. The most common concern of parents/caregivers was their ability to keep up with schoolwork. The QOL of participants who preferred to keep their epilepsy a secret (69) and those who used magazines and books (71) as one of the sources of information was better than that of those who did not.

Conclusion

Better QOL was found in males, adolescents, patients with higher family income, those living in Al Jubail, who preferred to keep their epilepsy a secret, and those who used magazines and books as sources of information. However, the QOL was negatively associated with seizure frequency, the number of fears, problems, and concerns that the patients/caregivers had, and longer treatment duration.

## Introduction

Approximately 50 million people of all ages suffer from epilepsy worldwide [1]. Epilepsy is a disease of the brain characterized by recurrent unprovoked seizures that result from abnormal electrical activity in the brain. Unprovoked seizures represent those without a known underlying cause [2]. Low and middle-income countries have a higher prevalence of epilepsy, mostly due to a higher prevalence of infections and trauma, which are risk factors for epilepsy [3]. Moreover, the incidence of epilepsy is expected to increase with time as life expectancy increases worldwide and also as advances in health care have resulted in the survival of patients who suffered incidents, predisposing them to develop epilepsy [1].

In Saudi Arabia, the prevalence rate of active epilepsy is 6.54/1,000 (95% CI 5.48-7.60), and most cases present as generalized onset [3-5]. In Saudi Arabia, the major neurological disorders are the most common chronic pediatric disorders, where the epilepsy prevalence rate is 8.8 per 10,000 [6], with males being more affected [7]. Many patients with epilepsy have coexisting physical or psychiatric comorbidities, which affect their quality of life (QOL) [1,8].

Several studies have been conducted to evaluate and assess the QOL of patients with epilepsy and to explore the main factors that significantly affect QOL in different populations. In the Netherlands, in 2002, a cross-sectional study found that psychological adjustment and coping distress, loneliness, and stigma perception appeared to contribute most significantly to the outcome of QOL as judged by the patients themselves, regardless of their physical status, while the onset, seizure frequency, and side effects of antiepileptic drugs did not significantly affect the QOL [9]. In 2013, another cross-sectional study conducted in a tertiary care teaching hospital in India concluded that many factors influence the QOL of people with epilepsy, and the type of drug therapy plays an important role; for instance, patients on monotherapy had a better QOL mainly because of fewer side effects [10].

Furthermore, a study assessing the QOL of epileptic patients compared with healthy people in Neyshabur, Iran, found that the mean scores of epileptic patients were lower than those of healthy individuals [11]. Few studies have highlighted the QOL of young adults and teenagers with epilepsy. According to a cross-sectional study that was initiated in 2005 in children and teenagers with epilepsy across 16 different countries, seizures in 65% of children and teenagers caused them to lose, on average, seven school days per year. The majority of respondents took epilepsy medications, and more than one-third of them had experienced side effects. In addition, more than one-third expected the conditions to hinder their lives in the future [12]. One of the studies conducted in Italy in 2014 concluded that epilepsy impairs all aspects of QOL of both children and their families, including job, leisure activities, peer relationships, and economy [13].

Assessing the patients’ QOL is essential to evaluating them functionally and emotionally and is an important aspect that should be evaluated in any comprehensive patient-centered health care. As shown in previous studies conducted in different countries, the QOL of epileptic patients differs from one area and culture to another, depending on several factors affecting QOL. Assessing the perceived impact and the QOL of patients with a specific disease can reflect the quality of the comprehensive health care provided to those patients and highlights the factors affecting them in other aspects to be considered. Hitherto, there is little research in the Kingdom of Saudi Arabia, specifically in the Eastern Province, aiming to assess the perceived impact of epilepsy on children and adolescents. Our study will cover this topic and analyze its results in an integrated manner.

## Materials and methods

This was a cross-sectional study, and the target population included patients visiting three major hospitals in the Eastern Province of Saudi Arabia. The hospitals were tertiary care hospitals with specialized clinics for pediatric epilepsy, which have neuroscience centers with experts specialized in pediatric neurology and also services specialized for pediatric neurology clinics.

A prescheduled online questionnaire was distributed among children (two to nine years old) and adolescents (10 to 19 years old) diagnosed with epilepsy and followed up in the epilepsy clinics. Patients with provoked seizures and a single episode of seizure were excluded.

The minimum required sample size was calculated to be 306 using Epi Info 7.0 (CDC, Atlanta, Georgia), assuming a 50% good QOL, with a precision of 5% at a confidence interval of 95%, given that the number of epilepsy patients in these clinical settings is approximately 1,500. Data collection started in August 2020 till January 2021.

The survey is a self-administered questionnaire with two parts: the first part was developed by the International Bureau for Epilepsy (IBE) and comprises 15 multiple-choice and open-ended questions [12]. The questionnaires were designed to gather information on the following key areas: demographics (town, age, gender, and family income), seizure frequency, medication for seizures and treatment duration, effects of epilepsy and medications, time missed from school, and perceptions about epilepsy, including reactions of other people, impact on QOL, concerns, fears, and aspirations for the future. This part had questions targeting children and young people with epilepsy (completed with or without the help of an adult) as well as questions targeting parents or caregivers.

The second part of the questionnaire assessed the QOL using the 12-item Short Form Survey (SF-12). Twelve questions were answered on a five-point Likert scale. The SF-12 questionnaire assessment provides scores on eight health domains: physical functioning, physical role, bodily pain, general health, vitality, social functioning, emotional role, and mental health. Physical functioning has limitations in daily life due to health problems. The physical role scale measures role limitations due to physical health problems. The bodily pain scale assesses pain frequency and interference with the usual roles. The general health scale measures individual perceptions of general health. The vitality scale assesses energy levels and fatigue. The social functioning scale measures the extent to which ill-health interferes with social activities. The emotional roles scale assesses role limitations due to emotional problems, and the mental health scale measures psychological distress. They were categorized into the physical component score (PCS) and mental component score (MCS). Because of the low response rate of two questions in the QOL assessment ("Does your health now limit you from climbing several flights of stairs?" and "Do problems with your work or other regular activities as a result of your physical health cause you to accomplish less than you would like?"), they were dropped from the analysis; the low response rate might be due to the inapplicability of the questions because of the patient’s age.

The part of the questionnaires to be filled by the patients was completed if they were above 12 years old and capable of answering the questions on their own. For children aged 12 years old or younger, or unable to write, the questionnaire was answered with the help of an adult, and the survey was distributed online among the patients and their caregivers after obtaining their consent.

This research did not receive any specific grant from funding agencies in the public, commercial, or not-for-profit sectors.

Ethical considerations

Confidentiality and anonymity of the subjects were maintained, and the information was used for research purposes only. Approval from the ethical review board of hospitals was arranged (Imam Abdulrahman Bin Faisal University [IRB-UGS-2020-01-191] and King Fahad Specialist Hospital-Dammam [EXT0369]) along with the contact data of patients.

Informed written consent was obtained from the patients or their parents/guardians for those less than 18 years old.

Statistical analyses

Statistical analyses were performed using SPSS version 21.0 (IBM SPSS Statistics, Armonk, NY), and two-tailed statistical significance was set at p < 0.05. Reverse coding was performed where necessary so that higher scores reflected a better QOL. The percentage of the highest possible score was then computed for QOL questions (the value obtained was multiplied by 100 and divided by five).

Frequency distribution and summary statistics (range, median, mean, and standard deviation) were used for the descriptive analysis. Differences in PSC or MCS among related factors were analyzed using independent samples t-test and ANOVA with further correction for pairwise comparisons. The chi-square test was used to test the association between categorical variables. Multivariate analysis of variance (MANOVA) was performed with PCS and MCS as dependent variables, and all factors studied in the bivariate analyses were independent factors.

## Results

Demographics

A total of 310 out of 845 patients with epilepsy completed the online questionnaire, with a response rate of 43.7%. Of the patients, 61.6% were males (Table 1). Regarding age, 48.4% were adolescents, and 51.6% were children. The majority of patients lived in Dammam (40.6%) and had a family income of Saudi riyal (SR) 5,000-10,000 (38.7%).

**Table 1:** Sociodemographic characteristics of epileptic patients from Eastern Province, Kingdom of Saudi Arabia (2021). ^†^ No treatment because the patient was newly diagnosed or there was no need for treatment because the patient was diagnosed with pseudoseizures. ^‡^ One patient may have more than one answer. GABA-A, gamma-aminobutyric acid type A.

		No. (n = 310)	%
Gender	Male	191	61.6
	Female	119	38.4
Age (years)	Children	160	51.6
	Adolescents	150	48.4
Family income (Saudi riyal)	Less than 5,000	87	28.1
	5,000-10,000	120	38.7
	More than 10,000	103	33.2
Residential city	Dammam	126	40.6
	Al Khobar	79	25.5
	Qatif	37	11.9
	Jubail	12	3.9
	Al-Ahsa	25	8.1
	Other eastern cities	19	6.1
	Other than eastern region cities	12	3.9
Seizure frequency	Once or more/week	100	32.3
	Once or more/months	39	12.6
	Once or more/six months	37	11.9
	Once or more/6-12 months	25	8.1
	Once/one year or more	71	22.9
	I do not know	38	12.3
Treatment type^‡^	Drugs	299	96.5
	Surgical management	23	7.4
	Vagus nerve stimulation	11	3.5
	Diet	23	7.4
	No treatment^†^	5	1.6
Number of drugs taken	Min-max, mean ± SD median	1-6, 1.64 ± 1 1	
Drug name^‡^	Valproate	121	39.0
	Levetiracetam (Keppra)	117	37.7
	Sodium channel blocking agents	103	33.2
	GABA-A inducers	52	16.8
	Topiramate	37	11.9
	Clozapine	23	7.4
	Vigabatrin	16	5.2
	Calcium channel blocking agents	6	1.9
	Primidone	2	.6
	Other	12	3.9
	I do not know	4	1.3
	No treatment	5	1.6
Treatment duration	One year or less	74	23.9
	2 to 3 years	100	32.3
	4 to 6 years	68	21.9
	6 to 12 years	51	16.5
	13 years or more	17	5.5
Side effect^‡^	Weight change	125	40.3
	Headache	73	23.5
	Dizziness	48	15.5
	Shaking	89	28.7
	Appetite change	3	1.0
	Imbalance	7	2.3
	Sleepiness	18	5.8
	No side effects	43	13.9

Seizure characteristics

Approximately one-third (32.3%) of children and adolescents reported one or more seizure episodes per week (highest seizure frequency), and almost 23% of children and adolescents have one seizure episode during one year or more (lowest seizure frequency) (Table 1).

Treatment and side effects

Almost all (96.5%) children and adolescents were treated with one or more antiepileptic medications alone or in combination with other treatment modalities (Table 1). Valproate and levetiracetam were the most frequently used drugs (39% and 37.7%, respectively). The treatment duration varied from one year or less in 23.9% to 13 years or more in 5.5% of the patients. Regarding side effects, weight change was the most reported side effect (40.1%). However, 13.9% of the patients did not develop any side effects. A small percentage of the patients reported sleepiness, imbalance, and appetite changes as side effects of their treatments. When asked about their preference to keep having epilepsy a secret, approximately two-thirds of the participants (64.8%) answered no.

Impact on life

Two-fifths (42.6%) of the patients were not in school/rehabilitation/educational or other similar centers, while 11.3% of the participants reported missing more than one day per week from school (Table 2). Approximately one-third of the epileptic children/adolescents (27.1%) miss at least two days or more per month. Although there is no wide variation between the percentages of concerns, the most common concerns children and adolescents with epilepsy have is about having/starting a relationship with others and what people at school will think if they have a seizure (22.6% and 21%, respectively); however, only 8% worried about being able to keep up with schoolwork. Of the participants, 58.4% could not answer the question about their concerns due to their age/condition. Approximately half of the patients had problems with concentration (49.7%) and feeling short-tempered or grumpy (48.4%). Regarding fears about the future, continuing with education is the most common fear among children with epilepsy. Patients reported driving, being diseased for the rest of their life, getting worse with time, dependency, and adapting/communicating with the community as additional future fears. The ability to keep up with schoolwork is the most common concern of parents/caregivers, accounting for more than half (65.2%) of them. The majority (85.5%) of the participants received information from their doctors or nurses, and one-half (55.5%) considered the internet as one of the sources of information.

**Table 2:** Perceived impact of epilepsy on life. ^†^ Not in school/rehabilitation/educational or other similar centers. ^‡^ One patient may have more than one answer.

impact		No. (n = 310)	%
Days missed from school	1 or more/week	54	17.4
	2 or more/month	30	9.7
	1 /month	22	7.1
	1/2-6 months	24	7.7
	1/7-12 months	11	3.5
	1/year	37	11.9
	Not applicable^†^	132	42.6
Areas of regular concern for the child/adolescent^‡^	I worry about having/starting a relationship with others	70	22.6
	I worry about what people at school will think if I have a seizure	65	21.0
	I avoid going on school trips	53	17.1
	I am less confident meeting with new people	46	14.8
	I find it difficult to make new friends	43	13.9
	I avoid trying new activities	37	11.9
	I worry about being able to keep up with schoolwork	25	8.1
	Does not apply (due to the age/condition)	181	58.4
Common problems they face^‡^	Lack of concentration	154	49.7
	Feeling short-tempered or grumpy	150	48.4
	Feeling sleepy	103	33.2
	Feeling muddled or confused	85	27.4
	Feeling sad or tearful	76	24.5
	Understanding instructions	65	21.0
	Feeling that you have no energy to do things	53	17.1
	Remembering things	51	16.5
	Describing things to other people	48	15.5
	Working out sums or other problems	45	14.5
	Working out how to do something new	39	12.6
	Writing or copying figures	27	8.7
Future fears concerning^‡^	Continuing with education	148	47.7
	Getting the job you want	128	41.3
	Having children	68	21.9
	Getting friends	55	17.7
	Traveling and exploring	43	13.9
	Being diseased for the rest of the life	16	5.2
	Adapt/communicate with the community	11	3.5
	Getting worse	6	1.9
	Dependency	6	1.9
	Driving	4	1.3
	No fears	9	2.9
	I do not know/NA	36	11.6
Areas of regular concern for parents/caregivers^‡^	Ability to keep up with schoolwork	202	65.2
	Reduced quality of life	136	43.9
	Learning new things	129	41.6
	Reduced self-esteem	128	41.3
	Joining in sport or other hobbies	123	39.7
	Going on school trips	94	30.3
	Making new friends	78	25.2
	Confidence to join new activities	73	23.5
Source of information about epilepsy	From doctors or nurses	265	85.5
	Internet	172	55.5
	books and magazines	31	10.0
	Family relatives	4	1.3
	Others experience	4	1.3

QOL assessment

**Figure 1:**
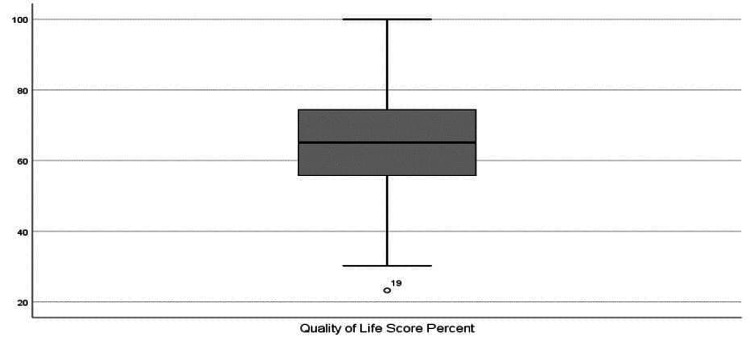
Quality of life score percent.

The mean score percentage of the QOL assessment was 65.6 (Figure 1). There was better QOL in males (67.10) than in females (63.08) (p = 0.026), and better QOL in patients living in Al Jubail (70.9). These differences were statistically significant as shown in Table 3. In addition, there was a higher QOL score percentage in adolescents (66.08) than in pediatrics (65.07) (p = 0.566) and patients with better family income (67.17) (p = 0.566); however, these differences were not statistically significant (Table 3).

**Table 3:** Quality of life in epileptic patients among different demographics and seizure characteristics. SR, Saudi riyal; GABA-A, gamma-aminobutyric acid type A.

		Minimum	Maximum	Mean	Standard deviation	Median	Test of sig	p-value
Gender	Male	23.26	100.00	67.10	14.90	67.44	t = 2.243	.026
	Female	30.23	100.00	63.08	16.01	62.79		
Age	Children	23.26	100.00	65.07	16.54	65.12	t = −.575	.566
	Adolescents	30.23	100.00	66.08	14.19	67.44		
Family income	Less than 5,000 SR	30.23	100.00	64.26	14.03	65.12	F = .919	.400
	5,000-10,000 SR	34.88	95.35	65.12	13.35	65.12		
	More than 10,000 SR	23.26	100.00	67.17	18.52	67.44		
City	Dammam	30.23	97.67	65.36	14.59	66.28	F = 2.926	.004
	Al Khobar	37.21	100.00	70.77	14.59	69.77		
	Qatif	23.26	88.37	62.23	16.04	60.47		
	Jubail	30.23	100.00	70.93	19.92	70.93		
	Al-Ahsa	30.23	83.72	61.30	15.01	60.47		
	Other cities in the Eastern Province	30.23	76.74	60.22	12.57	62.79		
	Northern region cities	32.56	72.09	53.49	16.22	54.65		
	Southern region cities	30.23	79.07	54.82	19.91	51.16		
	Western region cities	69.77	69.77	69.77	.	69.77		
Seizure frequency	Once or more/week	23.26	95.35	57.88	14.74	58.14	F = 10.756	.000
	Once or more/month	39.53	95.35	63.27	13.53	62.79		
	Once or more/6 months	37.21	90.70	69.52	12.75	72.09		
	Once or more/6-12 months	58.14	100.00	75.35	10.70	72.09		
	Once per 1 year or more	39.53	97.67	69.80	13.32	69.77		
	I do not know	30.23	100.00	69.89	18.77	70.93		
Drug name	Valproate	30.23	100.00	65.00	15.71	65.12	Each category is compared to non-users of this drug using a t-test	.611
	Levetiracetam (Keppra)	32.56	95.35	66.91	13.53	67.44		.232
	Primidone	39.53	41.86	40.70	1.64	40.70		.022
	Vigabatrin	23.26	76.74	50.00	13.61	52.33		.000
	Topiramate	30.23	83.72	60.28	12.82	60.47		.026
	Clozapine	30.23	83.72	58.65	14.62	58.14		.025
	Sodium channel blocking agents	30.23	97.67	64.53	15.16	65.12		.408
	Calcium channel blocking agents	44.19	95.35	61.63	18.41	59.30		.530
	GABA-A inducers	23.26	95.35	54.92	15.87	54.65		.000
	Other	30.23	79.07	56.59	15.66	56.98		.040
	I do not know	37.21	62.79	54.07	12.07	58.14		308
	No treatment	51.16	93.02	71.16	16.98	72.09		.414
Treatment duration	1 year or less	30.23	100.00	69.1075	16.19246	72.09	F = 3.496	.008
	2 to 3 years	23.26	100.00	66.7907	16.30114	66.28		
	4 to 6 years	39.53	97.67	65.3557	13.44161	66.28		
	6 to 12 years	30.23	86.05	59.3707	13.50723	58.14		
	13 years or more	30.23	88.37	62.2435	15.17273	62.79		
Do you prefer to keep your epilepsy a secret?	No	23.26	97.67	63.74	15.60	65.12	F = −2.851	.005
	Yes	30.23	100.00	68.91	14.60	67.44		

Potential associated factors with QOL

QOL was negatively correlated with the number of drugs used (r = −0.3, p = 0.0001). As shown in Table 4, days missed from school were negatively correlated with QOL (p = 0.000), which was statistically significant. Moreover, there was a significant negative correlation between treatment duration and QOL score percentage, where the patients treated for one year or less had the highest QOL score percentage, while it was the lowest for those treated for six to 12 years, as shown in Table 3. There was also a significant negative correlation between seizure frequency and QOL score percentage. Better QOL is associated with children and adolescents who had no side effects in comparison with those who had at least one of the side effects. The highest QOL is associated with using magazines and books as one of the sources of information, and worse QOL is associated with using others’ experiences, but this is not statistically significant. In addition, higher QOL was associated with those who were treated with the diet compared with drugs, surgical intervention, and vagus nerve stimulation; however, this was not statistically significant (Table 5). There was a negative correlation between QOL and the number of patients’ concerns (r = −0.142, p = 0.012), number of problems faced by the patients (r = −264, p = 0.0001), number of future fears (r = −0.248, p = 0.0001), and parents/caregivers concerns (r = −0.322, p = 0.0001), as shown in Table 4.

**Table 4:** Quality of life and epilepsy vs. impact on life.

		Quality of life score percent
Number of days missed from school	r	−.370
	p	.0001
Concerns	r	−.142
	p	.012
Problems	r	−.264
	p	.0001
Future fears	r	−.248
	p	.0001
Parents/caregiver's concerns	r	−.322
	p	.0001
Seizure frequency	r	−.233
	p	.0001
Drugs	r	−.305
	p	.0001

**Table 5:** Quality of life in epileptic patients and side effects, source of information, and treatment. Multiple response variables. Results are based on two-sided tests, adjusted for all pairwise comparisons using the Bonferroni correction.

		Minimum	Maximum	Mean	Standard deviation	Median	p-value
Side effect	Weight change	23.26	100.00	62.57	15.42	62.79	<0.05
	Headache	32.56	93.02	63.59	14.03	65.12	
	Dizziness	30.23	95.35	59.93	14.57	59.30	
	Shaking	23.26	100.00	60.26	14.54	58.14	
	Appetite change	39.53	55.81	47.29	8.17	46.51	
	Imbalance	39.53	74.42	57.48	13.81	62.79	
	Sleepiness	39.53	95.35	63.57	15.43	67.44	
	Other	30.23	97.67	64.81	16.03	65.12	
	No symptoms	30.23	95.35	73.77	15.99	74.42	
Source of information	Internet	30.23	100.00	64.94	15.30	65.12	>0.05
	Doctors and nurses	23.26	100.00	65.53	16.01	65.12	
	Books and magazines	30.23	100.00	71.19	16.71	72.09	
	Family relatives	30.23	72.09	60.47	20.18	69.77	
	Others experience	44.19	58.14	50.00	6.15	48.84	
	Other	69.77	72.09	70.93	1.64	70.93	
Treatment	Surgical management	34.9	90.7	64.3	14.1	67.4	>0.05
	Drugs	23.3	100.0	65.2	15.3	65.1	
	Vagus nerve stimulation	37.2	95.4	63.9	18.0	65.1	
	Diet	30.2	100.0	65.5	18.9	65.1	
	No treatment	51.2	93.0	71.2	17.0	72.1	

## Discussion

Age and gender

This study aimed to assess the QOL and survey the perceived impact of epilepsy and its treatment on children and adolescents with epilepsy in the Eastern Province of Saudi Arabia, some of which are consistent with those of previous studies conducted in different countries. The sample was relatively balanced between boys and girls (ratio of boys to girls was 1.6:1) and between the two age groups of children and adolescents (0.93:1), similar to the IBE sample, where there was no significant difference between the gender and the age group in the sample [12]. Our study shows that boys and adolescents have better QOL than girls and children. However, in Nadkarni et al.'s study, which assessed the QOL using Quality of Life in Childhood Epilepsy (QOLCE) questionnaire of 102 children with epilepsy aged five to 15 years in India, 59.8% were boys and 61.76% were children aged five to nine years old; the study concluded that girls with epilepsy had a better QOL score (62.01) than males (60.39). Overall QOL was affected more in older children than in younger age groups [14]. In addition, Devinsky et al.'s study on adolescents in Canada used QOLIE-AD-48 and reported poorer QOL in older adolescents (aged 14-17 years) [15]. This may be due to the social and cultural differences, as there exist more availability, opportunity, and choices of joining most outdoor activities for older children and boys in the Saudi Arabian society. The positive relation between the impact of epilepsy and time expended in extracurricular activities is also shown in Devinsky et al.'s study [15]. Cultural factors may also play a role in the lower QOL of girls because females receive less attention and care than males in some households. In addition, girls may show different sensitivities to the QOL domains than boys, which might explain the differences in the QOL by gender.

Family income and city

In this study, the sample included a similar proportion of each family’s income and socioeconomic level (28.1%, 38.7%, and 33.2%), and there was a significant difference between their QOL. Moreover, those in Al Jubail had the best QOL. While the mean difference in the QOL score percentage between Al Jubail and Al Khobar is only 0.161, the difference ranged between 5.5 and 10.7 when Al Jubail is compared to other Eastern Province cities, and 15.3 (p = 0.013) when compared to other cities outside the Eastern Province. Al Jubail and Al Khobar are important modern industrial cities in Saudi Arabia, characterized by their advanced administrative systems, business continuity, entertainment places, and the good socioeconomic status of their inhabitants. These special characteristics may be the reason behind the better QOL in comparison to other cities, in addition to the effect of the presence of medical facility access variations, illiteracy, negative attitudes, and stigma in rural areas. Another explanation for the better QOL in Al Jubail and Al Khobar might be the number of residents in households; for example, Dammam and Al-Ahsa have a larger population than Al Jubail and Al Khobar [16], which may mean that more residents live in a house, which affects the quality of care provided to the child or adolescent by the family. The impact of living area and socioeconomic status on QOL has also been reported in studies by Nadkarni et al. and Devinsky et al. [14,15].

Seizure frequency

Regarding seizure frequency, approximately the same percentages were reported by the IBE study and ours, where approximately two-thirds of the patients reported uncontrolled seizures (more than once per year) [12]. In general, this study shows that seizure frequency is negatively related to QOL, which is consistent with previous studies [15].

Treatment and drug name

In this study, higher QOL was associated with those treated with diet compared with drugs, surgical intervention, and vagus nerve stimulation; however, this difference was not statistically significant. Moreover, among those treated with drugs, the highest QOL was seen with levetiracetam, with a mean of 66.91, and the lowest was seen with Primidone, with a mean of 40.70, but neither of them was statistically significant. In a study conducted in the USA, patients on carbamazepine showed worse emotional functioning, whereas patients on valproic acid showed better emotional functioning [17]. In our study, there was no major difference between sodium channel blockers, including carbamazepine and valproic acid, with 64.53 and 65.00, respectively.

Drug number

Polytherapy is associated with more concerns and a significantly poorer QOL [18]. In another study, the mean of antiepileptic drugs was 2, with a standard deviation of 1.1, and those on polytherapy had worse psychosocial health when compared to children on monotherapy (p < 0.004) [19].

The results of the previous studies are in line with the findings of the current study, with a significant Pearson correlation of −0.305 (p = 0.000).

Treatment duration

A longer duration of treatment was significantly associated with a poorer QOL (r = 0.36) [18], which is consistent with the findings of another study in which children with longer treatment durations were more likely to have a greater impact on life and more concerned parents [20]. The findings of our study are consistent with those of previous studies because the highest mean of QOL was among those on treatment for a year or less (69.10), and lowest among patients on treatment for six to 12 years (F = 3.496, p = 0.008).

Side effects

In this study, better QOL was associated with children and adolescents who did not complain of any side effects (m = 73.77), with a significance of less than 0.05. Moreover, among those who had side effects, worse QOL was observed in patients complaining of imbalance (m = 57.48), but this was not statistically significant. The side effects of antiepileptic medications negatively affect health-related QOL in the physical, emotional, social, and school functions [17], consistent with the findings of other studies conducted in the UK, where the side effects and QOL were studied over time, and more side effects predicted worse health-related QOL subscale scores [21]. Stepwise regression analysis results of a study conducted in the USA showed that the number of side effects and QOL in children was R2 = 0.57 (p < 0.01) [22].

Fears and problems

Despite the healthcare services and cultural and social variations of the studied populations in this study and the IBE study, continuing education and getting a job they want were the most common future fears reported by participants in this study; similarly, in the IBE study, the previously stated fears were two of the most common fears. In addition, more than one-third of the patients reported problems with concentration and feeling short-tempered or grumpy in both samples [12]. The negative relationship between the number of fears and problems with the QOL that was found in this study may indicate the negative psychological impact of having many fears and problems on the QOL, or even the tendency of patients with low QOL to have more fears and problems. The significance of the psychological and social aspects of QOL in epileptic children has also been reported in previous studies [14].

Concerns

The most-reported concerns by parents were regarding the possible medical effects of seizures on the child, the effects of the antiepileptic drugs, concerns about the future, and parenting [18]. Parents’ stress was negatively associated with predictors of behavior, attention/concentration, memory, language, and self-esteem [21]. In a study exploring the concerns of epileptic children and adolescents and their parents, the mean number of concerns for parents was 4.7, while it was 3.6 for children [23]. The results of a stepwise regression analysis conducted in the USA also showed that greater parental anxiety was associated with a poor QOL (R2 = 0.50, p < 0.01) [22]. In our study, the number of concerns by patients was weakly correlated but significant with the QOL score percentage (−.142, p = 0.012), and the number of parents’ concerns was moderately correlated and statistically significant (−.322, p = 0.000).

Source of information

Our study showed that the highest QOL was significantly associated with using magazines and books (m = 71) as one of the sources of information when compared with those who did not use them. However, the worst QOL was associated with using others’ experiences (m = 50), but this was not statistically significant. The results of a cross-sectional study conducted in Gabon comparing the QOL of children receiving medical advice from general practitioners and specialists showed a QOL of 28 in those consulting general practitioners and 11 in those consulting specialists, but the difference was not statistically significant [24].

Keeping epilepsy a secret

MacLeod and Austin reviewed the literature on how keeping epilepsy a secret affects the QOL in different studies. Westbrook et al. reported that the majority of patients kept their epilepsy a secret, and the higher the level of perceived stigma, the poorer the self-esteem [25]. Moreover, Cramer et al.’s study included patients from Canada and the USA following up at epilepsy centers and showed a strong correlation between stigma and QOL (r = 0.64). The adolescents in this study reported relatively low levels of stigma and higher QOL. Additionally, Dunn et al.’s cross-sectional study among adolescents showed a low and significant correlation between stigma and depression (r ¼ 0.26 and 0.28) [25]. Another study by Westbrook et al. compared the tendency to keep the medical condition a secret among adolescents with epilepsy and adolescents with other chronic diseases; those with epilepsy were more likely to keep their condition a secret [25]. The QOL of our participants who preferred to keep their epilepsy a secret was better than that of those who did not (F = −2.851, p = 0.005); this might be due to a decreased perception of stigma, knowing that others do not know about their conditions and having more chance to be involved in all activities with their peers without special considerations.

## Conclusions

This study aims to assess the impact of epilepsy and its treatment on the QOL, development, and opportunities for children and adolescents with epilepsy in the Eastern Province of the Kingdom of Saudi Arabia. It showed a better QOL in boys and adolescents, patients with higher family income and socioeconomic level, and those living in Al Jubail. However, the QOL was negatively associated with seizure frequency, the number of fears, problems, and concerns that the patients/caregivers had, and longer treatment duration. The most common concerns in children and adolescents with epilepsy were having/starting a relationship with others, and what people at school will think if they have a seizure. The most common problems were problems with concentration and feeling short-tempered or grumpy. Continuing with education is the most common future fear. The most common concern of parents/caregivers was the ability to keep up with schoolwork. The QOL in participants who preferred to keep their epilepsy a secret and those who used magazines and books as one of the sources of information was better than those who do not.

Limitation

WHO defines pediatrics as those below 19 years; the study followed this definition in designing the study methodology and questionnaire and presenting the results of this study. However, hospital policies in Saudi Arabia differ from some hospital policies of other countries, usually pediatric departments where our sample follow up offer medical services for those aged 14-16 years old and younger, which made it difficult to recruit a good number of participants aged older than 14-16 years in the study from pediatric clinics. For that reason, the QOL and perceived impact of adolescents who were older than the age of 14-16 years could be underestimated in the studied sample.

Application

These findings highlight several issues and factors affecting children and adolescents with epilepsy in different aspects of their life. As such, these findings should be considered to provide more comprehensive health care to these patients to facilitate the movement toward improving their health and developing best health practices and care in the near future.
